# Quasi-3D Model for Lateral Resonances on Homogeneous BAW Resonators

**DOI:** 10.3390/mi14111980

**Published:** 2023-10-25

**Authors:** Carlos Udaondo, Carlos Collado, Jordi Mateu

**Affiliations:** Signal Theory and Communications (TSC) Department, Universitat Politècnica de Catalunya (UPC), 08034 Barcelona, Spain; carlos.udaondo@upc.edu (C.U.); jordi.mateu-mateu@upc.edu (J.M.)

**Keywords:** BAW resonator, Film Bulk Acoustic Resonator, Lamb wave, solidly mounted resonator, spurious modes

## Abstract

Lateral modes are responsible for the in-band spurious resonances that appear on BAW resonators, degrading the in-band filter response. In this work, a fast computational method based on the transmission line matrix (TLM) method is employed to model the lateral resonances of BAW resonators. Using the precomputed dispersion curves of Lamb waves and an equivalent characteristic impedance for the TE_1_ mode, a network of transmission lines is used to calculate the magnitude of field distributions on the electrodes. These characteristics are specific to the stack layer configuration. The model’s implementation is based on nodal Y matrices, from which particle displacement profiles are coupled to the electric domain via piezoelectric constitutive relations. Consequently, the input impedance of the resonator is obtained. The model exhibits strong agreement with FEM simulations of FBARs and SMRs, and with measurements of several SMRs. The proposed model can provide accurate predictions of resonator input impedance, which is around 200 times faster than conventional FEM.

## 1. Introduction

The advent of 5G networks has raised the bar for acoustic filters in RF front-ends, necessitating a deeper understanding of the spurious resonances in resonators. Spurious modes, particularly their cancellation, are essential considerations in BAW resonator design [1,2,3]. These spurious modes are primarily associated with the lateral propagation of Lamb waves within a resonator [4], which can be employed for designing novel resonant structures [5]. However, for devices like FBARs and SMR-BAW, these lateral effects pose significant challenges [6].

To model those lateral effects, finite element method (FEM) simulations are commonly used. Two-dimensional FEM simulations are used to analyze the Lamb modes and their dispersion curves within a stack [7]. However, when aiming to simulate the behavior of an in-plane arbitrarily shaped resonator, three-dimensional FEM simulations are mandatory [8]. Despite the accuracy of FEM simulations, it is worth noting that they demand substantial memory and computational power, particularly for solidly mounted resonators (SMRs) with complex layer configurations [8].

In order to model those lateral effects, finite element method (FEM) simulations are mostly used. Two-dimensional simulations are performed to obtain a stack’s Lamb modes and their dispersion curves [7]. However, when aiming to simulate the behavior of an in-plane arbitrarily shaped resonator, three-dimensional FEM simulations are mandatory [8]. Despite the reliability of three-dimensional FEM simulations, the computational requirements are extremely heavy, especially for solidly mounted resonators (SMR) with many layers in their stack [8].

Over the past decade, various models have been developed to address the challenges associated with simulating spurious modes and their impact on second harmonic emissions (H2) in BAW filters. These models include Mason-based approaches [9,10,11] and analytical solutions for two-dimensional simulations [12,13]. 

One advantage of these models is their computational efficiency compared to that of the FEM. However, they are primarily suitable for modeling simple in-plane geometries. The influence of these spurious modes on second harmonic emissions (H2) has been a critical factor in BAW filter design, leading to the development of several models that consider these lateral resonances [14,15,16,17]. Many of these models, however, rely on other methods, e.g., fitting spurious resonances to a measurement, to determine the characteristics of lateral resonances.

In a recent study [18], a two-dimensional scalar wave model was introduced to enable the modeling of arbitrary in-plane geometries. This model uses the scalar wave equation to determine the motional values for each mBVD branch. Additionally, in another publication [19], a model utilizing the transmission line matrix (TLM) method was demonstrated by our group for modeling the lateral dimensions of a square SMR. This research expands upon the previous work, allowing for the simulation of various in-plane geometries.

The primary objective of this article is to introduce a fast computational model capable of delivering consistent results across different types of BAW resonator technologies, regardless of their in-plane geometry. To meet this objective, several essential criteria must be fulfilled:

- The parameters used to model the dispersive behavior of the resonator must be independent of the resonator’s in-plane geometry;
- The model should adopt a discretized approach focusing solely on the lateral dimensions to minimize the number of unknowns;
- The model must be adaptable to simulate multi-layered structures, even though it models only the lateral dimensions.

Using the TLM method and extracting stack-dependent variables, including dispersive behavior, our proposed model can calculate the in-plane field distribution of particle velocities. This distribution is instrumental in determining the input impedance of BAW devices with varying in-plane geometries.

The article is structured as follows. In Section 2, a brief overview of Lamb waves and their equivalent transmission lines is given to the reader. The curve fitting of the required dispersive curve is discussed and compared with the one provided for 2D FEM simulations of FBAR and SMR resonators.

The model for two-dimensional resonators is presented in Section 3. The results are then compared with 2D FEM simulations of film bulk acoustic resonators (FBARs) and SMRs. In Section 4, the work is expanded to the third dimension and compared with 3D FEM simulations and measurements of SMRs.

## 2. Lamb Waves and Their Equivalent Transmission Line

On a homogeneous isotropic plate, longitudinal (L) and transverse vertical (TV) waves are sagittal-polarized and propagate along the lateral dimensions. On plate-free surfaces, both waves are coupled, thus generating a set of dispersive modes [20].

### 2.1. Lamb Waves

The displacement distribution of a plate with a thickness, *t*, defined in the *z* direction, that is infinite in the *y* direction, and a wave propagating in the *x* direction becomes the following [20]:

(1) $$\begin{array}{l} u_{x}=\left(qA\cos\left(qz\right)-jkB\cos\left(pz\right)\right)e^{\mp jkx}\\ u_{z}=\left(jkA\sin\left(qz\right)-pB\sin\left(pz\right)\right)e^{\mp jkx}, \end{array}$$

where *p* and *q* are, respectively, defined as *p*^2^ = *ω*^2^/*V_L_*^2^ − *k*^2^ and *q*^2^ = *ω*^2^/*V_T_*^2^ − *k*^2^, *V_L_* and *V_T_* being the phase velocities of the longitudinal and transverse vertical waves, respectively. The wavenumber (*k*) of the Lamb wave will follow the Rayleigh–Lamb equation:

(2) $$\frac{\omega^{4}}{V_{T}^{4}}=4q^{2}k^{2}\left(1-\frac{p\tan\left(pt/2+\alpha \right)}{q\tan\left(qt/2+\alpha \right)}\right).$$

where the constant *α* becomes zero for symmetric modes and *π*/2 for antisymmetric ones. Antisymmetric modes do not couple to the electric response of BAW resonators [4], so they are not going to be considered in this work.

**Figure 1:**
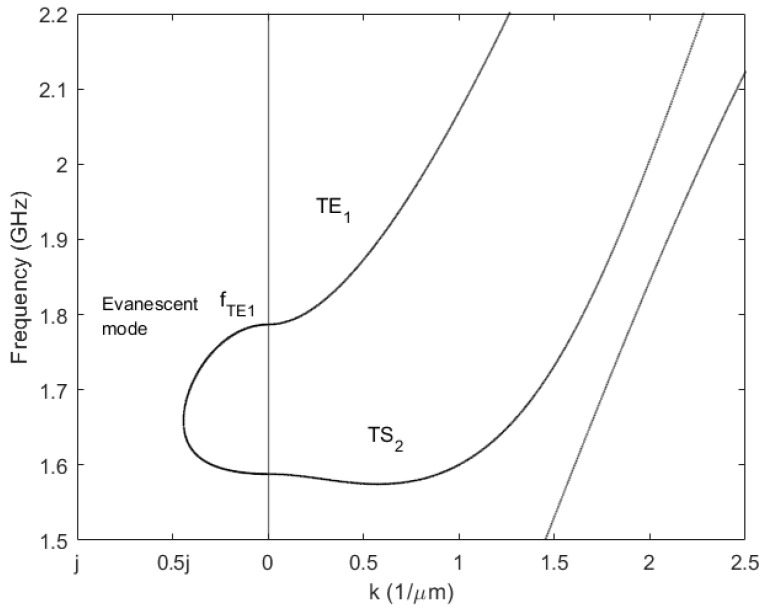
Dispersion curves of a 1.74 μm thickness ZnO plate. The curves for two symmetric modes TE_1_, TS_2_, and the evanescent mode, between their cutoff frequencies, are labeled on the plot.

Equation (2) is the dispersion relation of the propagating wave. The dispersion relation for a Lamb wave has no analytical solution and numerical methods like FEM are needed to solve it [7]. In Figure 1, the dispersion curves of a ZnO piezoelectric membrane exhibiting Type I dispersion are calculated with COMSOL.

The TE_1_ mode is the one responsible for the lateral resonances on a BAW resonator [4]. In an infinite plate, the TE_1_ mode propagates along the lateral dimensions, but on a BAW resonator, the acoustic energy is confined in the electrode region [21]. The reflection of the acoustic waves on the lateral boundaries generates the lateral resonances that appear on the resonator’s impedance [4].

### 2.2. Field Magnitudes of the TE_1_ Mode

For low wavenumbers (*k*~0), and with TE_1_ being the first odd symmetric mode (*A*~0), its displacements become the following [20]:

(3) $$u_{x}\approx 0;\;u_{z}=-pB\sin\left(pz\right)e^{\mp jkx}.$$

The tangential stress, *T_xz_*, will have the same distribution on the thickness direction than the displacement, *u_z_*:

(4) $$T_{xz}=c_{55}\left(\pm jkpB\right)\sin\left(pz\right)e^{\mp jkx},$$

where *c*_55_ is one of the constants of the material stiffness tensor.

The field magnitudes *u_z_* and *T_xz_* are the ones that determine the propagating modes that laterally resonate, generating the spurious resonances that can be modeled using the TLM method.

#### 2.2.1. Equivalent Transmission Line

An electrical analogy of the acoustic wave is used to develop a transmission line model [19]. The equivalences between acoustic and electric transmission lines relate the mechanical force (*F*) with the electric voltage (*V*), and the particle velocity (*v_z_*) with the electric current intensity (*I*). 

**Figure 2:**
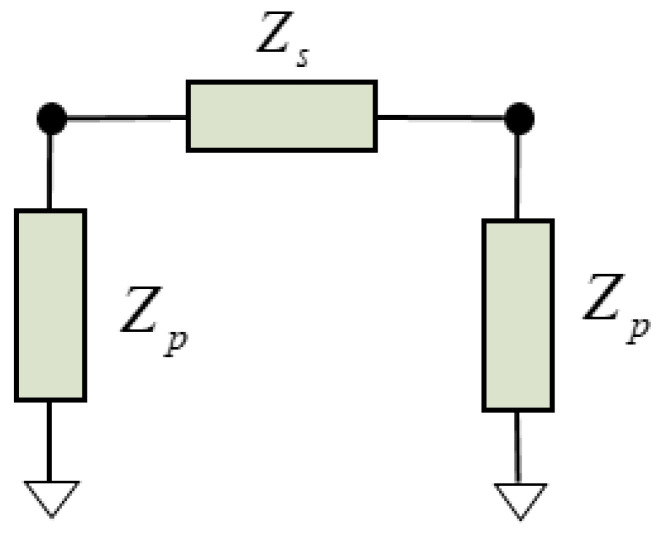
Two-port *Π*-network for a transmission line.

The conventional transmission line for modeling the TE_1_ mode is a two-port *Π*-network (Figure 2) with

(5) $$Z_{s}=jZ_{0}\cdot \sin\left(\beta l\right)$$

(6) $$Z_{p}=-jZ_{0}\frac{1}{\tan\left(\beta l/2\right)},$$

where *l* is the physical length of the transmission line. The phase constant (*β*) and the characteristic impedance (*Z*_0_) need to be defined for the TE_1_ mode.

#### 2.2.2. Phase Constant

In order to model the dispersive behavior of the TE_1_ mode, the phase constant needs to satisfy the Rayleigh–Lamb relation (2), so *β* = *k*. An analytic expression can be approximated to the precomputed dispersion curve for Type I resonators [9]:

(7) $$\beta =\frac{2\pi }{c_{lamb}}{\left(f^{r}-f_{001}^{r}\right)}^{1/r},$$

where the exponent *r* is close to 2, the constant *c_lamb_* is the estimated wave velocity of the mode, and *f*_001_ is the cut-off frequency of the TE_1_ mode, the so-called piston mode. 

**Figure 3:**
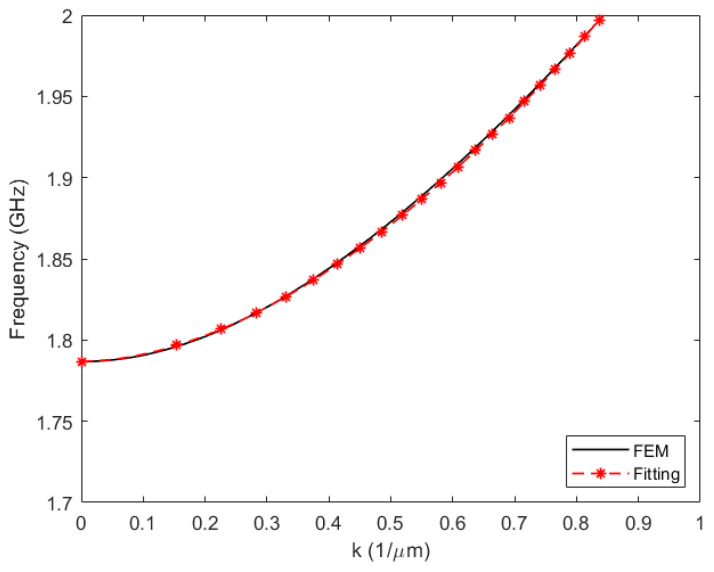
Dispersion curve for the TE_1_ mode of a 1.74 μm thickness ZnO plate. FEM simulations were performed to obtain the dispersion curve and then expression (7) was adjusted.

The subscript 001 indicates the number of half-wavelengths in the directions *x*, *y*, and *z*. Here, the 0 subscript indicates that the fields are uniform in the *x* and *y* directions, while the 1 subscript indicates half-wavelength on the thickness direction. The constants, *c_lamb_*, *r* and *f*_001_, are fitted to the measured or pre-calculated dispersion curve given a resonator’s stack (Figure 3).

For a rectangular resonator, one can find the resonating frequency of each mode as follows [9]:

(8) $$f_{mn1}^{r}={\left(c_{lamb}\frac{m}{2a}\right)}^{r}+{\left(c_{lamb}\frac{n}{2b}\right)}^{r}+{\left(f_{001}\right)}^{r},$$

where *a* and *b* correspond to the lateral dimension of the resonator. Here, the subscripts *m* and *n* (*m*, *n* = 1, 2, 3…) indicate the number of half-wavelengths of the mode in each lateral dimension.

#### 2.2.3. Characteristic Impedance

For a wave propagating in an acoustic transmission line, the characteristic impedance will relate the force (*F_z_*) and the particle velocity (*v_z_*) as [19] follows:

(9) $$F_{z}=Z_{0}\cdot v_{z},$$

(10) $$F_{z}=\int_{V}\frac{\partial T_{xz}}{\partial x}dV=T_{xz}A_{tb},$$

where *A_tb_* is the resonator’s cross-sectional area. Applying (3) and (4) with the definitions above, the characteristic impedance for the TE_1_ results in the following:

(11) $$Z_{0}=\frac{1}{\omega }\beta c_{55}A_{tb}.$$

#### 2.2.4. Low-Loss Transmission Line

Losses are introduced in the model by means of a complex propagation constant (*γ* = *α* + *jβ*), where α and *β* are the attenuation constant and the phase constant, respectively. Assuming that *α* << *β*, the Z_0_ and the phase constant remains the same as that in the lossless case.

## 3. Quasi-2D Model

**Figure 4:**
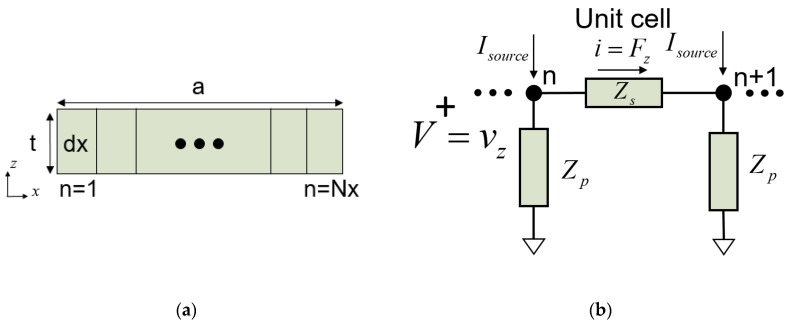
(**a**) Two-dimensional resonator in the *xz* plane. The *x* dimension is discretized in *N* elements; (**b**) equivalent *Π* network of a dispersive transmission line in the *x* direction.

In a two-dimensional resonator in the *xz* plane, as outlined in Figure 4a, the Lamb wave propagates in the *x* direction. The lateral dimension, *a*, is discretized in *N_x_* elements of length *dx*. 

### 3.1. Unit Cell

Each of the unit cells is modeled with the dispersive transmission line in Figure 2. Since we are using nodal Y matrices, in order to implement the stress-free lateral boundary conditions (see Section 3.2, below) [19], it is more convenient to invert the typical equivalences between electric and acoustic magnitudes. This means defining *V* as velocities and *I* as forces. Then, *Z*_0_ of the transmission line is defined as the inverse of the following (11):

(12) $$Z_{0}^{\prime }=a_{kz}\frac{\omega }{\beta \cdot c_{44}\cdot A_{tb}},$$

Here, *a_kz_* acts as a scaling factor to adjust resonator coupling. This is required because the displacement field (3) is a simplification obtained from the Lamb wave equations for non-piezoelectric isotropic plates [9]. 

The equivalent circuit of a unit cell of the TL is depicted in Figure 4b, where the values of the *Π* network are now as follows:

(13) $$Z_{s}=Z_{0}^{\prime }\cdot \sinh\left(\gamma \cdot dx\right)$$

(14) $$Z_{p}=Z_{0}^{\prime }\frac{1}{\tanh\left(\gamma \cdot dx/2\right)}$$

(15) $$dx={a/N}_{x}.$$

The propagation along the lateral dimension of the resonator is modeled cascading several unit cells.

### 3.2. Lateral Boundary Conditions

In an ideal resonator, the acoustic wave is reflected at its lateral edges, thus generating each of the lateral spurious resonances.

#### 3.2.1. Free Stress

The velocity, *v_z_*, at the boundaries will be zero, implying the full reflection of the acoustic waves at the lateral interfaces. To impose this boundary condition, the lateral transmission lines are ended with a short circuit. This way, the voltage at the end of the lines becomes zero.

#### 3.2.2. Lateral Leakage

Acoustic energy may leak out of the resonator’s active area. This effect is called lateral leakage and degenerates the *Q* factor around the antiresonance frequency [4]. If required, this effect can be modeled adding a conductance to the ends of the transmission line. 

### 3.3. Model Implementation

Applying an electric potential (*U*) on the resonator electrodes, a constant, *F_z_*, is generated along the resonator’s lateral dimension [22,23]. Due to the impedance inversion, it is modeled as distributed current sources (*I_source_*) at each cell node as follows:

(16) $$I_{source}=e\cdot dx\cdot b/t\cdot U,$$

where *e* is the piezoelectric constant for the piston mode, *b* is the lateral dimension on the *y* axis, and *t* is the thickness between the electrodes.

The distributed transmission line will be assembled in a nodal Y matrix made of (13) and (14). This matrix will have the dimension *N* × *N*, and will be excited with the current sources (16):

(17) $$\left[\begin{array}{ccccc} Y_{s}+Y_{p} & -Y_{s} & 0 & 0 & \ddots \\ -Y_{s} & Y_{s}+2\cdot Y_{p} & -Y_{s} & \ddots & 0\\ 0 & -Y_{s} & \ddots & -Y_{s} & 0\\ 0 & \ddots & -Y_{s} & Y_{s}+2\cdot Y_{p} & -Y_{s}\\ \ddots & 0 & 0 & -Y_{s} & Y_{s}+Y_{p} \end{array}\right]\cdot \left[\begin{array}{c} V_{1}\\ \vdots \\ V_{N} \end{array}\right]=\left[\begin{array}{c} I_{source}\\ \vdots \\ I_{source} \end{array}\right].$$

Once the system is solved, the velocities, *v_z_*, are obtained for each node along the lateral dimension, *a*. As a result, electrical current intensity, *I*, is generated between the electrodes due to each electromechanical contribution, as follows:

(18) $$I=\left(e\cdot dx\cdot b/t\right)\cdot \sum_{1}^{N}v_{z}\left(n\right),$$

and the acoustic contribution of the resonator’s admittance is found using (19):

(19) $$Y_{lat}=I/U.$$

To obtain the total resonator impedance, the static capacitance (*C*_0_) and the series resistance (*R_s_*) to model the Ohmic losses on the electrode [24] are included, resulting in the following:

(20) $$Z_{in}=R_{s}+\frac{1}{Y_{lat}+j\omega C_{0}}.$$

### 3.4. Comparison with FEM Simulations

A simple ZNO membrane of a thickness of 1.74 µm and with a lateral dimension *a* equal to 80 µm is simulated in 2D using COMSOL. Losses are kept artificially low to achieve more pronounced spurious modes. The dispersive curves of the membrane are obtained with COMSOL following the procedure described in [7], and the curve corresponding to the TE_1_ mode is fitted using the function described in (7) as is shown in Figure 3.

Using the obtained dispersive phase constant, with *f*_001_ = 1.787 GHz, *c_lamb_* = 6745, and *r* = 1.955, the lateral dimension of the resonator is modeled with *N_x_* = 80. This level of discretization is enough to ensure the convergence of the model for these resonators. The boundary conditions are set on the two extremes of the lateral line, shorting the nodes of the Y matrix that correspond to the boundaries. We refer to these kinds of simulations as quasi-2D simulations, since we are modeling only propagation in one direction, and the characteristics of the propagation mode along the thickness direction are considered using the dispersive phase constant and the associated characteristic impedance of the TE_1_ mode.

**Figure 5:**
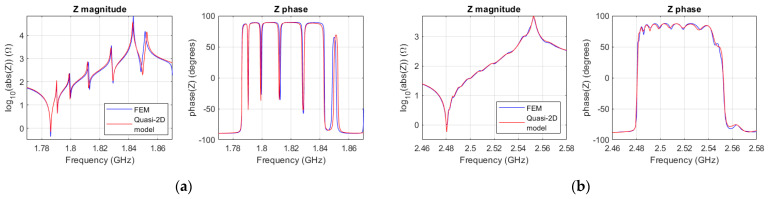
(**a**) Magnitude and phase of the impedance of the 2D FEM ZnO resonator (blue), and the quasi-2D model (red); (**b**) magnitude and phase of the impedance of the 2D FEM AlN SMR (blue), and the quasi-2D model (red).

Figure 5a shows the fitting of the quasi-2D model to the FEM simulation. The scaling of the characteristic impedance was fine-tuned with *a_kz_* = 0.9. This can be justifiable because the characteristic impedance was derived for an isotropic layer. The electric permittivity (*ε_r_*) and the piezoelectric constant are the same than that provided by COMSOL for ZnO. The attenuation constant of the dispersive line was set to *α* = 2000 Np/m.

The model was also tested simulating an AlN SMR resonator. FEM 2D simulations were performed with realistic losses in all layers in this case. The resonator has a top electrode comprising Al, W layers, a SiN passivation layer, a bottom electrode of W and Al layers, and a Bragg reflector comprising alternating SiO_2_ and W layers on a Si substrate. Its series resonance is around 2.48 GHz, and the *k*^2^*_eff_* is about 6.8%.

The dispersion curve was fitted with *f*_001_ = 2.48 GHz, *c_lamb_* = 5740, and *r* = 2.05, and the attenuation constant was tuned to *α* = 7800 Np/m. FEM simulations unveiled displacements outside the active area at frequencies near the antiresonance frequency. These losses (lateral leakage) were modeled with a conductance of 0.3 mS connected at the boundary nodes. The fitting of the Quasi-2D model can be seen in Figure 5b. In this case, the characteristic impedance was scaled using *a_kz_* = 0.5.

## 4. Quasi-3D Model

**Figure 6:**
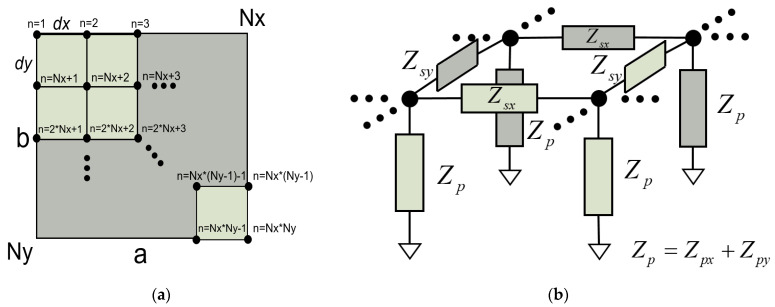
(**a**) Schematic of a TLM mesh for a square resonator. The lateral dimensions *a* and *b*, and the number of discretizations in each direction (*N_x_* and *N_y_*) are indicated; (**b**) equivalent *Π* network of a dispersive transmission line in the *x* and *y* direction. Four nodes interconnected by different directions of transmission lines are shown.

The Quasi-2D model can be extended to the other in-plane dimension using the transmission line matrix (TLM) method [25,26,27]. We refer to this model as Quasi-3D model. The TLM method appears as a discrete implementation of Huygens’ principle, where the waves propagate along a mesh of transmission lines connected by nodes (Figure 6a) [25]. When a node of the TLM mesh is excited, the energy spreads isotropically from the excited node. All the scattered fields along the mesh combine to form the overall waveform.

### 4.1. Unit Cell

To add the other dimension, an additional transmission line is added. The new transmission line will have the same dispersive behavior, and the length of the lateral dimension, *b*, will be discretized in *N_y_* elements of length *dy* = *b*/*N_y_*. The cross-sectional area will be different for each propagating direction. It ends up being *A_tb_* = *dy*·*t* for the *x* direction, and *A_ta_* = *dx*·*t* for the *y* direction. Note that this would allow the simulation of resonators with non-uniform shapes.

In Figure 6b, four nodes of the TLM mesh are shown. The impedances for each dimension are calculated considering *dx*, *dy*, and the different cross-sectional areas.

### 4.2. Losses and Boundary Conditions

Losses and boundary conditions are set as in the Quasi-2D model. A complex propagation constant is used in each propagation direction, and all the boundary nodes are shorted; if not, lateral leakage is considered or otherwise set with the correspondent conductance. 

### 4.3. Implementation of the Model

The resulting nodal Y matrix has a size of (*N_x_*·*N_y_*)^2^, where *N_x_* and *N_y_* are the number of discretizations along the *x* and *y* direction, respectively. The node numbering is illustrated in Figure 6b for the sake of clarity. 

The linear system of equations, an extended version of (17) will be solved. However, note that the excitation will be slightly different than the one described in (16), since now we must consider the discretization of the *y* direction (*dy*), which results in the following:

(21) $$I_{source}=e\cdot dx\cdot dy/t\cdot U.$$

Once the linear system is solved, the electric current generated between the electrodes is calculated as follows:

(22) $$I=\left(e\cdot dx\cdot dy/t\right)\cdot \sum_{1}^{N}v_{z}\left(n\right).$$

Finally, the electric input impedance of the resonator is calculated using (20), as is carried out for the quasi-2D model.

### 4.4. Quasi-3D Model vs. 3D FEM Simulations

A 3D ZnO membrane with the same thickness as that in the 2D case was simulated using COMSOL 6.0. Three different resonators with different in-plane geometries were simulated: a square resonator with an area of 80 × 80 µm^2^, a rectangular resonator with an area of 80 × 100 µm^2^, and a trapezoidal resonator with a height of 80 µm and two bases with heights of 40 µm and 80 µm, respectively. Losses are artificially kept much lower than those in a real resonator to show highly coupled modes.

The phase constant and characteristic impedance of the TE_1_ mode remains the same as that previously used in the quasi-2D model (*f*_001_ = 1.787 GHz, *c_lamb_* = 6745, *r* = 1.955, *a_kz_* = 0.9, and *α* = 2000 Np/m) as they are assumed to be characteristics of the stack. The stress-free lateral boundary conditions are applied at each edge node. 

**Figure 7:**
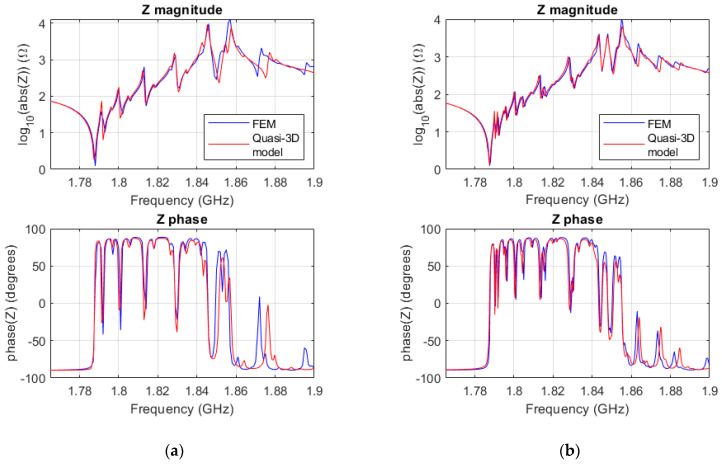

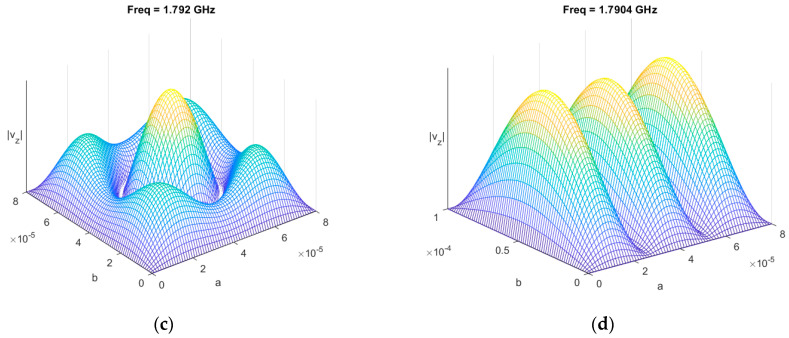
(**a**) Magnitude and phase of the impedance of the 3D FEM ZnO square resonator (blue), and the quasi-3D model (red); (**b**) magnitude and phase of the impedance of the 3D FEM ZnO rectangular resonator (blue), and the quasi-3D model (red); (**c**) standing wave pattern of |*v_z_*| at the frequency of mode 311 and 131 in the square resonator; (**d**) standing wave pattern of |*v_z_*| at the frequency of mode 311 in the rectangular resonator.

Figure 7a,b show the electrical input impedances for the square and rectangular resonators, respectively.

Comparing Figure 5a and Figure 7a, one can notice that although both are for the same square resonator, the latter has additional spurious modes. In the 2D model, constant displacement in the y direction of the resonator was assumed, and only *m*01 modes were simulated, in both the FEM simulation and the quasi-2D model. Adding the extra dimension, *y*, allowed the simulation of *mn*1 modes with an *n* different to zero. Those are the additional spurious modes that appear in Figure 7a. In a square resonator, since *a* = *b*, *mn*1 and the *nm*1 are degenerated modes, meaning that they occur at the same frequency. As an example, Figure 7c shows the resulting standing wave pattern of *v_z_* of the modes sharing the same frequency, modes 311 and 131.

Notice that the rectangular resonator (Figure 7b) shows more spurious modes, since *a* ≠ *b*, and therefore, the *mn*1 and the *nm*1 modes do not fall at the same frequency. In this case, and just for illustrative purposes, Figure 7c shows the *v_z_* of mode 311, showing three half wavelengths in the *x* direction and one in the *y* direction.

**Figure 8:**
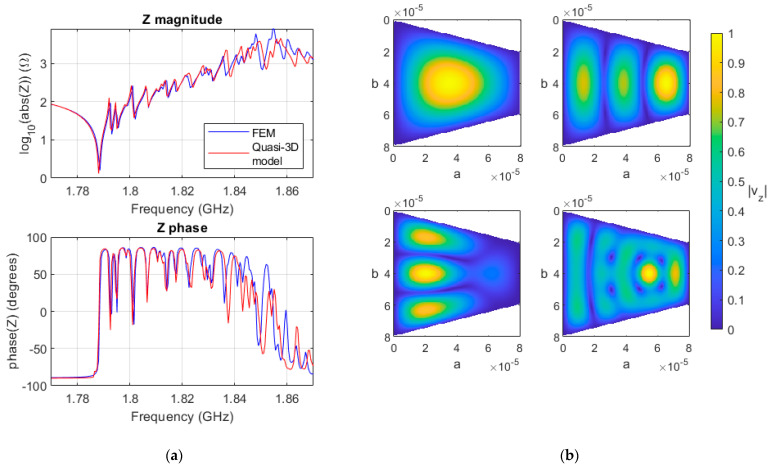
(**a**) Magnitude and phase of the impedance of the 3D FEM ZnO trapezoidal resonator (blue), and the quasi-3D model (red); (**b**) standing wave pattern of normalized |*v_z_*| for the first four resonant modes of the trapezoidal resonator.

The electrical impedance of a trapezoidal resonator is shown in Figure 8a. The quasi-3D model shows good agreement with FEM modeling. The number of discretization has to be increased from that of 80 × 80 used on the square and rectangular resonator to that of 180 × 180 to achieve convergence.

The first four resonant modes of the trapezoidal resonator can be observed in Figure 8b. The fundamental mode and the ones exhibiting three half-wavelengths in the larger dimensions can be clearly identified. The other mode cannot be defined as is usually carried out in rectangular resonators since it is a combination of the scatterings produced at the non-parallel interfaces.

### 4.5. Computational Time

Since the thickness dimension of the resonator does not have to be simulated, the number of DoFs is significantly reduced.

The computational time for both the FEM simulations and for our proposed model are presented in Table 1. They were calculated using the same number of frequencies and achieved mesh convergence in both cases. 

For the quasi-2(3)D model, an additional FEM simulation needs to be performed to obtain the stack characteristics. However, a 2D simulation of around 100 frequency points is enough to obtain the dispersive curve and electromechanical coupling.

The computational time is reduced by several orders of magnitude between the FEM and the TLM. The 2D SMR case shows a higher reduction due to the increase in DoFs in the FEM simulations. This is mainly due to the discretization along the thickness dimension with many layers involved. Although the number of unknowns for modeling any arbitrary electrode shape, like the trapezoidal one, is increased, the time needed to perform the quasi-3D simulation continues to be much lower than that required for FEM simulations.

Note that, as can be seen in Table 1, the number of unknowns is independent of the resonator layers, making this method very suitable when working with SMR, since the same number of nodes are needed as that for the FBARs.

We want to outline that the performance of the quasi-3D method has room to be upgraded, since 90% of the computational time in Matlab was taken by the assembling of the Y matrices. In addition, any symmetry was considered when using the TLM model;meanwhile, in FEM simulations, symmetries where used when possible to reduce the amount of computational resources needed. The symmetry strategy is also noted in Table 1.

## 5. Evaluation of Quasi-3D Simulations and Measurement Results

Four different SMR measurements were provided by the manufacturer of the devices for validating the model. The resonators have the same stack composition as does the FEM-simulated SMR. The resonators are made of two different areas (6400 µm^2^ and 12,900 µm^2^), and two different aspect ratios (1 and 2). 

The dispersion curve was fitted with *f*_001_ = 2.48 GHz, *c_lamb_* = 5180, and *r* = 2.2. However, the cut-off frequency of each resonator was slightly tuned (in the order of 100 ppm) to consider the tolerances of each resonator’s layers. The other material constants were adjusted to *e* = 1.51, and *ε_r_* = 9.8.

The propagation constant was set to *α* = 7800 Np/m, and lateral leakage was modeled by means of a conductance in the lateral boundaries. Since in a 3D geometry the lateral boundaries are discretized by *N_x_* and *N_y_*, the conductance connected at each node will be also divided by *N_x_* or *N_y_*. The conductance value used to fit the four resonators was 0.3/*N_i_* mS.

**Figure 9:**
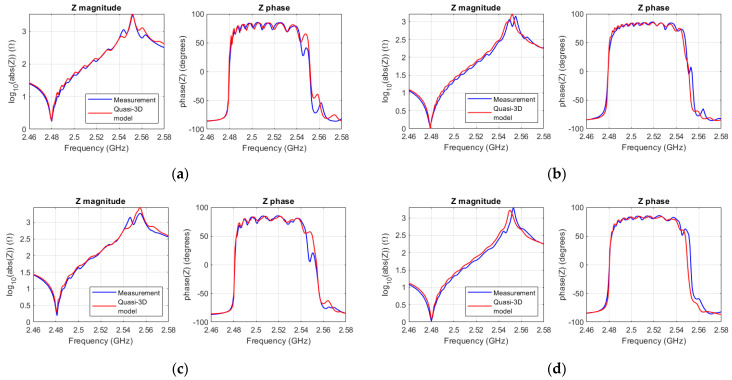
(**a**) Magnitude and phase of the impedance of the measured square SMR (A = 6400 µm^2^) (blue), and the quasi-3D model (red); (**b**) magnitude and phase of the Impedance of the measured square SMR (A = 12,900 µm^2^) (blue), and the quasi-3D model (red); (**c**) magnitude and phase of the impedance of for the measured rectangular SMR (A = 6400 µm^2^) (blue), and the quasi-3D model (red); (**d**) magnitude and phase of the impedance of for the measured rectangular SMR (A = 12,900 µm^2^).

Figure 9 displays the fitting of the quasi-3D model to four different measurements. The model exhibits strong agreement with the measurements near the fundamental frequency but displays slight deviations near the antiresonance. These deviations can likely be attributed to minor tolerances within each resonator stack, which prevent a single curve from precisely matching that of all four of the different resonators.

The computational time required for quasi-3D SMR simulations is the same as that for FBARs (refer to Table 1).

## 6. Conclusions

The validity of the TLM method for BAW devices was demonstrated through exhaustive comparisons with FEM simulations, including both 2D-FEM simulations and 3D-FEM simulations. These FEM simulations were performed using a commercial software, COMSOL Multiphysics 6.0, which is a FEM software widely used by stack developers and designers of electro-acoustic devices. 

Our proposed method offers a significantly faster computational time compared with that of the traditional methods such as FEM. This faster computational time streamlines the design process for BAW resonators, particularly by enabling the rapid simulation of lateral modes. The key concept is that by capturing the dispersive propagation constants of Lamb wave modes, either through 2D-FEM simulations or actual measurements of test resonators (typically square or rectangular in shape), we can include this dispersive characteristic (defined by the stack of materials) in transmission lines. The TLM method discretizes these lines solely in the in-plane direction. 

The TLM method has been applied to electromagnetic cavities for many years with the capability to handle various geometries. Its application in acoustics is promising, providing designers the ability to run optimizers in search of geometries that minimize the impact of spurious modes. 

Our method is implemented through a nodal Y matrix, creating a system of linear equations that can be solved using any standard software. The primary limitation is that the analysis is restricted to only one propagating mode, in this case the TE1 mode of the resonator, which is the main responsible for the spurious resonances on BAW devices.

When simulating apodized in-plane geometries, other limitations are found at the boundaries. Since we assume rectangular unit-cells, the boundaries at oblique edges are defined as step-by-step edges. This implies performing the simulations with a finer mesh that the one that would be required for rectangular resonators, for example. This could be improved using adaptive meshes that are finer at the edges and coarser at the central part of the resonators. This improvement has not been achieved yet, since this article is a proof-of-concept of the method. Nevertheless, it outperforms FEM simulations in terms of computational resources. Ensuring that the application of the TLM method to BAW resonators is promising and can provide designers the ability to run optimizers to find a better geometry that reduces the effect of the spurious modes.

The authors are currently in the process of implementing this method for modeling non-homogenous electrode BAW devices, i.e., resonators with a border ring. The ability to predict the cancellation of these spurious modes could also help to reduce the iterations of manufacturing test resonators with optimum in-plane geometries.

We anticipate that this approach could also be explored for simulating other types of acoustic resonators, including leaky SAW, TC-SAW, X-BAR, and others where lateral resonances are a factor. Naturally, dispersive curves should be calculated, and electroacoustic coupling should be defined accordingly.

## Figures and Tables

**Table 1:** Comparison of computational times between FEM and the proposed models.

Simulation	FEM (Time [DoF])	Quasi-2(3)D Model (Time [DoF])
ZnO membrane 2D (½ symmetry)	3 min 22 s [9300]	0.134 s [80]
SMR 2D (½ symmetry)	11 min 4 s [163,391]	0.134 s [80]
ZnO membrane 3D (¼ symmetry)	1 h 52 min [215,628]	42 s [6400]
ZnO trapezoidal 3D (½ symmetry)	2 h 55 min [630,752]	4 min 52 s [32,400]

## Data Availability

The data are not publicly available due to confidentiality.
